# Evaluating the Efficacy and Safety of CyberKnife for Meningiomas: A Systematic Review

**DOI:** 10.7759/cureus.56848

**Published:** 2024-03-24

**Authors:** Abdulrahman Bin Sumaida, Nandan M Shanbhag, Khalid Balaraj

**Affiliations:** 1 Oncology/Radiation Oncology, Tawam Hospital, Al Ain, ARE; 2 Internal Medicine, College of Medicine and Health Sciences, United Arab Emirates University, Al Ain, ARE; 3 Oncology/Radiation Oncology/Palliative Care, Tawam Hospital, Al Ain, ARE

**Keywords:** brain stereotactic radiosurgery, robotic stereotactic radiotherapy, treatment of meningiomas, meningiomas, cyberknife® radiosurgery

## Abstract

This systematic review aims to evaluate CyberKnife (Accuray, Madison, WI, USA) radiosurgery's efficacy, safety, and outcomes in treating meningiomas, focusing on tumour control rates, symptom relief, survival rates, quality of life, and adverse events. A comprehensive literature search was conducted across PubMed, EMBASE, Web of Science, Google Scholar, and Cumulative Index to Nursing and Allied Health Literature (CINAHL), covering studies published in the last 20 years and available in English. The inclusion criteria targeted studies involving patients with meningioma treated with CyberKnife radiosurgery, reporting on specific outcomes of interest. Quality assessment was performed using the Newcastle-Ottawa Scale for observational studies, and a narrative synthesis approach was adopted for data analysis. Twenty-one studies met the inclusion criteria, encompassing various design types and patient demographics. The review highlights CyberKnife's effectiveness in managing benign and atypical meningiomas and specific challenging cases like perioptic lesions and large cranial base tumours. Key findings include high tumour control rates, preservation or improvement of visual functions in perioptic lesions, and promising results in benign spinal tumours and supratentorial meningiomas. Comparative analyses suggest better radiographic tumour control and a lower incidence of post-treatment complications with stereotactic radiotherapy over stereotactic radiosurgery. Long-term outcomes and safety profiles underline the viability of CyberKnife as a treatment option, with minimal permanent side effects reported. CyberKnife radiosurgery is a highly effective and safe treatment modality for meningiomas. It offers significant benefits in tumour control, symptom relief, and maintaining the quality of life with minimal adverse effects. The precision and adaptability of CyberKnife technology make it a valuable addition to the treatment arsenal for meningiomas. It necessitates further research and adoption in clinical practice, especially in regions like the United Arab Emirates, where its use is emerging.

## Introduction and background

Meningiomas are adults' most common primary brain tumours, comprising about 20% to 30% of all intracranial tumours [1]. They originate from the meninges, the membranes that surround the brain and spinal cord. The prevalence of meningiomas generally increases with age. It is more common in females, with a female-to-male ratio of approximately 2:1. The overall incidence of meningiomas is about 2.3 to 7.8 per 100,000 people, with the incidence rate rising in older populations [2]. Most meningiomas are benign (WHO grade I). Still, there are also atypical (WHO grade II) and anaplastic (WHO grade III) meningiomas, which show more aggressive behaviour and a higher risk of recurrence [3].

Several risk factors have been identified for the development of meningiomas [4]. Ionizing radiation is a well-established risk factor, and hormonal factors are also implicated due to the higher prevalence in females and hormone receptors in some tumours. Other potential etiological factors include genetic predispositions, such as mutations in the neurofibromatosis gene (NF2), and environmental exposures [5].

Meningiomas exhibit various clinical presentations depending on their anatomic locations [6]. Symptoms and clinical syndromes associated with meningiomas vary, making familiarity with their diverse clinical manifestations crucial for accurate diagnosis. The clinical presentation of meningiomas can range from headaches and visual disturbances to more specific neurological deficits, such as cranial nerve palsies, depending on the tumour's location and size [7]. For instance, meningiomas in the pediatric age group can present with seizures, and their clinical features might differ significantly from adult cases, indicating the need for a tailored approach to diagnosis and management across different age groups [8]. Unique cases, such as meningiomas presenting with psychiatric symptoms or mimicking conditions like puerperal psychosis, highlight the complexity and variability of their clinical manifestations [9]. The broad spectrum of symptoms associated with meningiomas signifies the importance of considering these tumours in the differential diagnosis of various neurologic and psychiatric presentations [10].

The diagnosis of meningiomas largely relies on radiological imaging, with magnetic resonance imaging (MRI) being the cornerstone in identifying and characterizing these tumours [11]. Modern imaging is crucial in the initial diagnosis, postoperative evaluation, and follow-up studies of meningioma patients. Imaging characteristics of meningiomas are typically diagnostic, although they can sometimes present atypically. Meningiomas are commonly present as extra-axial tumours with a broad dural base. They may exhibit a 'dural tail' sign on contrast-enhanced MRI scans, indicating dural infiltration or vascular supply from the dura. They generally show homogenous enhancement with contrast, reflecting their vascularity [12]. The imaging appearance can vary depending on the tumour's histological subtype and location. Still, some common features include hyperostosis of the overlying skull, calcifications within the tumour, and potential brain oedema surrounding the tumour. Computed tomography (CT) scans can be particularly useful in identifying calcifications and bone changes associated with meningiomas. However, MRI provides superior soft-tissue contrast and detail, making it more effective in assessing tumour extent, involvement of adjacent structures, and potential differential diagnoses [13].

Advances in radionics and artificial intelligence in medical imaging are beginning to provide objective and quantitative approaches to interpreting imaging data, offering potential insights beyond traditional visual observations. These advances could further improve the diagnostic accuracy and prognostic prediction for meningioma patients, aiding in treatment planning and outcome prediction [14].

The primary treatment for meningiomas involves surgical resection, which can be curative if the tumour is completely removed [15]. Gross-total resection should be aimed at the parasagittal, lateral sphenoid wing, and olfactory groove meningiomas. More conservative surgical approaches may be employed to preserve neurological function for tumours located at the skull base or those involving vital brain structures. Embolization before surgery may reduce intraoperative bleeding and prevent postoperative complications [16]. In cases where surgery is not feasible or for residual or recurrent tumours, radiotherapy, including stereotactic radiosurgery and fractionated radiotherapy, is commonly used [17]. Radiotherapy effectively reduces recurrence rates with limited toxicity, especially for atypical or malignant meningiomas, which are best treated with fractionated radiation therapy with conventional margins [18].

Chemotherapy has shown modest activity and is generally reserved for selected cases. The most commonly recognized medical therapies for inoperable and radiation-refractory meningiomas include hydroxyurea, interferon (IFN-α), and octreotide long-acting release (LAR), a somatostatin analogue. However, the effectiveness of these treatments remains limited, and there is an ongoing need for more effective systemic treatments [19]. Recent advances in the molecular understanding of meningiomas have paved the way for novel therapeutic opportunities. Identifying mutations such as Neurofibromatosis Type 2 (NF2), Smoothened (SMO), Telomerase Reverse Transcriptase (TERT), TNF Receptor-Associated Factor 7 (TRAF7), and methylation profiling provide new insights into prognosis and treatment options. Although early results have been modest, targeted molecular therapies are being explored in clinical trials. Angiogenesis inhibitors and other targeted agents inhibiting specific cell signalling pathways show promise for future treatment strategies [20].

CyberKnife (Accuray, Madison, WI, USA) stereotactic radiosurgery (SRS) and hypofractionated stereotactic radiotherapy (hSRT) have emerged as significant advancements in treating meningiomas, providing a non-invasive alternative or adjuvant to conventional surgery and radiotherapy [21]. CyberKnife offers precise, high-dose radiation therapy targeting the tumour, sparing surrounding normal tissues [22]. This approach is particularly beneficial for treating meningiomas near the organs at risk (OAR), where surgical intervention carries a high risk of morbidity [23]. Despite the growing body of evidence supporting its use, there remains a need for a comprehensive synthesis of the available data to elucidate the true efficacy and safety profile of CyberKnife treatment in meningioma patients. This systematic review aims to bridge this knowledge gap by rigorously evaluating and summarizing the current evidence on the outcomes of CyberKnife radiosurgery for meningiomas. By doing so, this review explores to provide a clear and evidence-based insight into its role in tumour control, symptom relief, survival rates, quality of life, and adverse events, thereby guiding clinicians in optimizing treatment strategies and improving patient care.

## Review

Methods

Search Strategy

A comprehensive search was conducted across several databases: PubMed, EMBASE, Web of Science, Google Scholar, and Cumulative Index to Nursing and Allied Health Literature (CINAHL), aiming to capture all relevant literature on the use of CyberKnife radiosurgery for meningioma treatment published in the last 20 years and available in English. The search strategy was designed to include terms related to "meningioma" and "CyberKnife" without combining them with other treatments to focus on tumour control rate, symptom relief, survival rate, quality of life, and adverse events (AEs). Specific search queries were tailored to each database's syntax and capabilities to ensure broad and accurate retrieval of articles. The searches were executed on February 21, 2024.

Selection Criteria

Eligibility criteria for the inclusion of studies in this review were rigorously predefined to ensure alignment with the specific objectives of the review. Studies were eligible for inclusion if they met the following criteria: publication within the preceding 20 years and in the English language, involvement of patients diagnosed with meningioma, evaluation of the efficacy of CyberKnife radiosurgery as a therapeutic intervention, and documentation of outcomes such as tumor control rate, symptom relief rate, survival rate, quality of life, and adverse events.

Conversely, exclusion criteria were meticulously established to maintain the integrity of the review's focus on the specific effects of CyberKnife radiosurgery. Studies were excluded from the review if they failed to isolate the effects of CyberKnife radiosurgery from other therapeutic interventions, or if they constituted case reports, comments, letters to the editor, or conference abstracts that lacked full-text availability.

Data Extraction

Two reviewers independently screened titles and abstracts for eligibility, followed by a full-text review to confirm inclusion. Discrepancies were resolved through discussion or consultation with a third reviewer. Data were extracted using a standardized form to capture study characteristics (author, year, study design), patient demographics, treatment specifics (dose, fractionation), and outcomes (tumour control, symptom relief, survival rate, quality of life, adverse events).

Quality Assessment

The quality of included studies was assessed using appropriate tools based on the study design, such as the Newcastle-Ottawa Scale for observational studies. This assessment focused on the selection of study groups, comparability of groups, and the ascertainment of outcomes of interest.

Data Synthesis and Analysis

Due to the anticipated heterogeneity of studies in design and outcomes, a narrative synthesis was planned. For studies not amenable to the review, findings were presented descriptively.

Ethical Considerations

**Figure 1:**
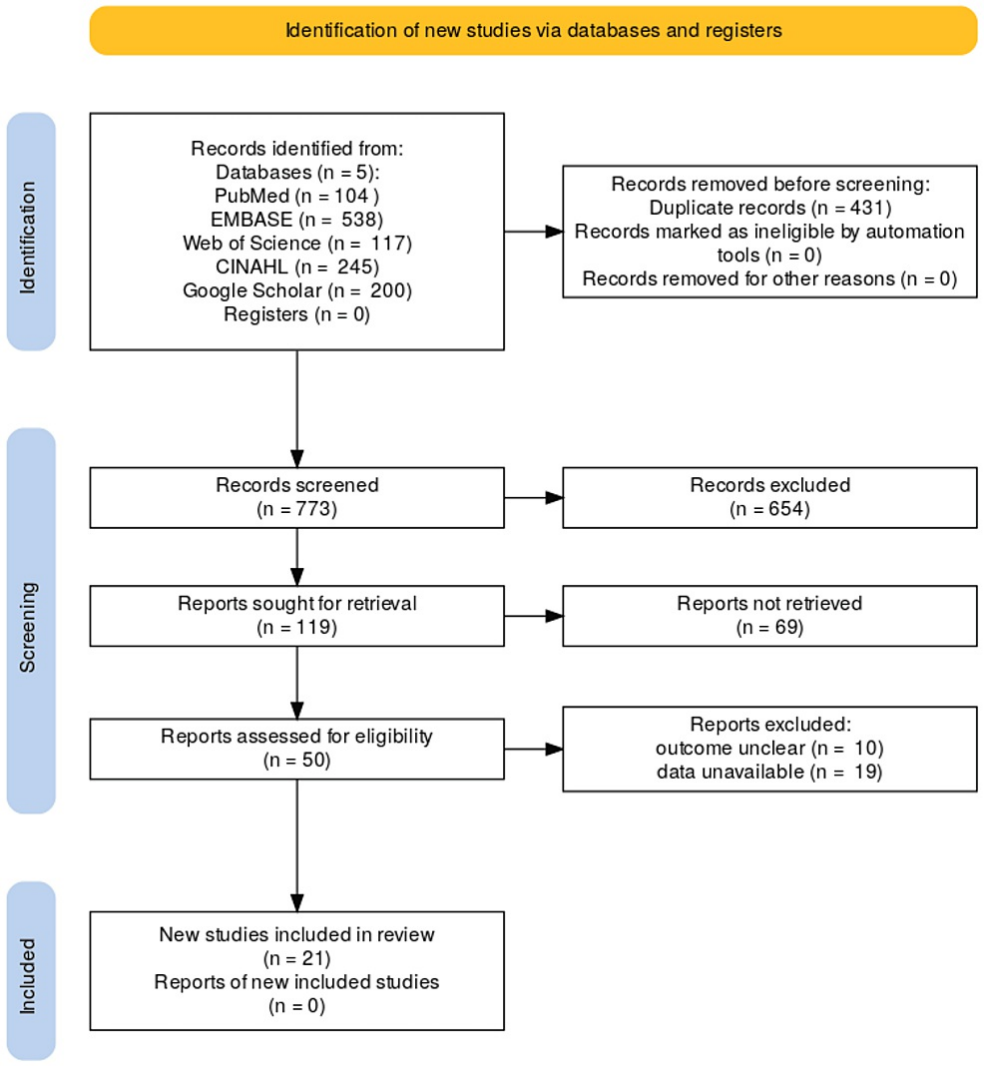
Preferred Reporting Items for Systematic Reviews and Meta-Analyses (PRISMA) flow diagram

This research did not require direct ethical approval as a systematic review of published studies. However, all processes were conducted in adherence to the Preferred Reporting Items for Systematic Reviews and Meta-Analyses (PRISMA) guidelines to ensure the integrity and transparency of the review [24]. The PRISMA flow diagram is depicted in Figure 1. 

This systematic review on using CyberKnife to treat meningioma encompassed a range of studies that varied in design, patient demographics, and outcomes. This detailed synthesis of the findings from the included studies, focusing on treatment methods, efficacy, safety, and long-term outcomes, is presented in Table 1. 

**Table 1:** Comparative analysis of outcomes in CyberKnife for meningiomas: a review of clinical studies SRS: Stereotactic Radiosurgery; CKRT: CyberKnife Radiotherapy; CSM: Cavernous Sinus Meningiomas; ICMs: Intracranial Meningiomas; ONSM: Optic Nerve Sheath Meningiomas; PM: Petroclival Meningiomas; PORT: Postoperative Radiotherapy; SRT: Stereotactic Radiotherapy; FU: Follow-Up; PFS: Progression-Free Survival; HSRT: Hypofractionated Stereotactic Radiotherapy; FSRT: Fractionated Stereotactic Radiotherapy; RRS: Robotic Radiosurgery

Authors	Year	Study Type	Objective	Methods	No. of Patients	Tumor Types	Treatment Details	Follow-Up Period	Results	Conclusion	Adverse Effects
Pham CJ, et al. [25]	2004	Retrospective Study	Treat perioptic lesions	34 patients with staged radiosurgery	34	Meningiomas, Pituitary Adenomas	2-5 stages, 20.0 Gy avg.	Mean 29 months	91% visual function preserved	Effective for lesions adjacent to optic apparatus	Visual loss in 3 patients
Adler JR Jr, et al. [26]	2006	Retrospective Study	Assess multisession radiosurgery	49 patients, perioptic tumours	49	Various perioptic tumours	2-5 sessions, 20.3 Gy avg.	Mean 49 months	Vision unchanged in 38, improved in 8	Effective for perioptic tumours	Visual deterioration in 3 cases
Sahgal A, et al. [27]	2007	Retrospective Review	Evaluate CyberKnife for benign spinal tumors	16 patients, 19 tumours	16	Various benign spinal tumours	Median 21 Gy in 3 fractions	Median 25 months	Local control acceptable, 3 progressions	Promising for benign spinal tumours	Progression in 3 tumors
Patil CG, et al. [28]	2008	Retrospective Study	Identify predictors of oedema post-SRS	102 patients with meningiomas	102	Supratentorial Meningiomas	Median 18.0 Gy in 1-5 fractions	Mean 20.9 months	14.7% developed symptomatic oedema	Parasagittal location increases the risk of oedema	Symptomatic oedema in 15 patients
Tuniz F, et al. [29]	2009	Retrospective Study	Report on large benign cranial base tumours	34 patients with large tumours	34	Various cranial base tumours	2-5 sessions, 24 Gy median	Median 31 months	All tumors locally controlled	Safe for large benign cranial base tumours	Neurological worsening in 4 patients
Choi CY, et al. [30]	2010	Retrospective Review	Review outcomes for atypical meningiomas	25 patients with atypical meningiomas	25	Atypical Meningiomas	Median 22 Gy in 1-4 fractions	Median 28 months	High control rates; predictors of recurrence	Effective for atypical meningiomas	Radiation toxicity in 2 cases
Morimoto M, et al. [31]	2011	Retrospective Study	Evaluate hypofractionated SRT	31 patients with meningiomas	31	Intracranial Meningiomas	21 to 36 Gy in 3-5 fractions	Median 48 months	87% 5-year progression-free rate	Volume indicative of oedema risk	Marked oedema in 6 patients
Oermann EK, et al. [32]	2013	Retrospective Review	Assess five fraction radiosurgery	38 patients	38	Benign Meningiomas	Median total 25 Gy	Median 20 months	Neurologic symptoms improved in 58.3%	Effective and well-tolerated	Minimal acute toxicity
Fatima N, et al. [17]	2019	Systematic Review and Meta-analysis	Compare SRS and SRT	Systematic review, 1736 patients	1736	Intracranial Meningiomas	Varied, including Gamma Knife and CyberKnife	Median 35.5 months	SRT showed better control	SRT preferable over SRS	Higher risk of worsening with SRS
Marchetti M, et al. [33]	2019	Retrospective Multicenter Study	Evaluate 25 Gy in 5 fractions for meningiomas	167 patients	167	Skull Base Meningiomas	25 Gy in 5 consecutive days	Median 51 months	High progression-free survival	Effective for meningiomas near AOP	Visual worsening in 3.7%
Terpolilli NA, et al. [34]	2019	Retrospective Study	Analyze outcomes after targeted resection	122 patients with orbital meningiomas	122	Orbital Meningiomas	Targeted resection followed by PORT or watch and wait	Mean 70 months	Longer progression in PORT group	Early PORT beneficial	Not specified
Liu J, et al. [35]	2020	Retrospective Review	Assess CKRT for olfactory groove meningiomas	13 patients	13	Olfactory Groove Meningiomas	SRS, HSRT, FSRT with median doses	Median 48 months	100% regional control rate	Safe and effective for OGMs	Edema requiring decompression in one patient
Oh HJ, et al. [36]	2020	Retrospective Study	Investigate hypofractionated SRS for large meningiomas	31 patients	31	Skull Base Meningiomas	5 daily fractions, 27.8 Gy median	Median 57 months	90.3% tumor control	Promising for large-sized skull base meningiomas	Not specified
Ruess D, et al. [37]	2020	Retrospective Analysis	Report on long-term FU of CSM treated with SRS	116 patients	116	Cavernous Sinus Meningiomas	Single fraction SRS, 12.6 Gy median	Median 55 months	98% tumor control at 2 and 5 years	Excellent control for CSM	Toxicity in 10.3% of patients
Hong S, et al. [38]	2021	Retrospective Analysis	Evaluate long-term effects of SRT on CSMs	113 patients	113	Central Skull Base Meningiomas	Median dose 25 Gy	Median 49 months	78% free of progression at 10 years	Useful for CSMs with low adverse events	Optic neuropathy and cerebral edema
Lovo EE, et al. [39]	2021	Retrospective Analysis	Analyze dosing for meningiomas focusing on dural tail	143 patients	143	WHO Grade I Meningiomas	Varied platforms, focus on dural tail coverage	Up to July 2020	96% tumour control	Dural tail coverage doesn't improve control	Grade 4 toxicity in one patient
Nguyen EK, et al. [40]	2021	Retrospective Review	Assess hSRT for benign ICMs	62 patients	62	Benign Intracranial Meningiomas	3-5 fractions, 18 Gy common	Median 64.7 months	85.2% 5-year PFS	Effective with acceptable toxicity	Grade III/IV toxicity in 3.2%, radionecrosis in 4.8%
Ruge MI, et al. [41]	2021	Retrospective Single-centre Analysis	Evaluate SRS for resectable meningiomas	188 patients	188	Intracranial Meningiomas	Single fraction SRS, 13.0 Gy avg.	Median 55.8 months	0.5% local recurrence	Effective for potentially resectable meningiomas	Early and late adverse events related to symptoms
Senger C, et al. [42]	2021	Retrospective Analysis	Evaluate RRS for ONSM	25 patients	25	Optic Nerve Sheath Meningiomas	4-5 fractions, 20.0-25.0 Gy	Mean 37.4 months	96.0% tumor control	Safe and effective for ONSM	Not specified
Wijaya JH, et al. [43]	2022	Systematic Review	Examine SRS in treating PM	Systematic review, 719 patients	719	Petroclival Meningiomas	Various SRS modalities and doses	Up to 252 months	46.5% tumor size decrease	Effective for PM with low complication rates	Hydrocephalus in 2.2%
Grzbiela H, et al. [44]	2023	Retrospective Study	Assess dose de-escalation in RRS for meningiomas	172 patients	172	Intracranial Meningiomas	18 Gy in three fractions	18 to 124 months	98.8% crude PFS after treatment	Effective comparable to higher doses	No late effects observed

Discussion

Analyzing the 21 studies provided, several key themes emerge regarding using CyberKnife in managing meningiomas [25-44].

Tumor Control and Visual Outcomes in Perioptic Lesions

Studies by Pham et al. (2004), Adler et al. (2006), and Marchetti et al. (2019) focused on the effectiveness of SRS and SRT in periodic lesions, demonstrating high rates of tumour control and preservation or improvement of visual function. Pham et al. reported that 91% of patients retained their presurgical vision following staged radiosurgery. In comparison, Adler et al. found that 94% of patients retained or improved their vision after multisession CyberKnife radiosurgery. Marchetti et al. further validated the effectiveness of multisession radiosurgery, with progression-free survival rates of 98%, 94%, and 90% at three, five, and eight years, respectively [25,26,33].

Management of Benign Spinal Tumors

Sahgal et al. (2007) evaluated using the CyberKnife Radiosurgery System to treat benign spinal tumours, finding acceptable local control with short follow-up. The study highlights the potential of CyberKnife spine stereotactic body radiotherapy (SBRT) for benign spinal tumours, reporting that three tumours progressed post-treatment, indicating a need for longer follow-up to assess long-term control better [27].

Effectiveness of SRS/SRT in Supratentorial Meningiomas and Large Cranial Base Tumors

Several studies addressed the efficacy of SRS/SRT in treating supratentorial meningiomas and large cranial base tumours. Patil et al. (2008) and Tuniz et al. (2009) highlighted the risk of peritumoral oedema following SRS, especially in patients with parasagittal meningiomas [28,29]. Tuniz et al. reported that multisession radiosurgery appears safe and effective for large benign cranial base lesions, with no permanent neurotoxicity observed within their follow-up period [28].

Management of Atypical Meningiomas and Specific Cases

Choi et al. (2010) and Liu et al. (2020) provided insights into treating atypical meningiomas and olfactory groove meningiomas, respectively. Choi et al. highlighted that irradiating the entire postoperative tumour bed might not be necessary for most patients with subtotally resected atypical meningiomas, achieving outcomes comparable to historical control rates for larger volume radiotherapy [30]. Liu et al. concluded that CyberKnife radiotherapy is safe and effective for treating olfactory groove meningiomas, with a 100% regional control rate and a significant reduction in tumour volume in their cohort [35].

Comparative Efficacy and Safety Analyses

Fatima et al. (2019) conducted a systematic review and meta-analysis comparing SRS and SRT's safety and long-term efficacy. They found that SRT provided better radiographic tumour control and a lower incidence of posttreatment symptomatic worsening and symptomatic oedema than SRS [17].

Long-Term Outcomes and Safety

Terpolilli et al. (2019), Oh et al. (2020), and Ruess et al. (2020) discussed the long-term outcomes and safety of SRS in the treatment of orbital meningiomas, large-sized skull base meningiomas, and cavernous sinus meningiomas [34,36,37]. They collectively emphasized the excellent long-term tumour and symptom control provided by SRS with minimal permanent side effects, suggesting SRS as a viable treatment option.

Adaptation to Local Clinical Practices

In the context of the United Arab Emirates (UAE) and broader Middle Eastern healthcare settings, integrating CyberKnife technology into local clinical practices could revolutionize the management of meningiomas and similar conditions. The detailed studies provided, such as those by Pham et al. (2004), Adler et al. (2006), and Marchetti et al. (2019), not only attest to the efficacy and safety of the technology but also signify the potential for improving patient outcomes through tailored treatment protocols. For instance, the high tumour control rates and preservation of visual function in perioptic lesions emphasize the technology's capability to target tumours with remarkable accuracy, minimizing damage to surrounding critical structures [25,26,33].

Considerations for Long-Term Implementation

For successful implementation in the Middle East, considerations must include training for local radiation oncology teams, adapting treatment protocols to suit the regional healthcare landscape, and evaluating long-term outcomes in the local patient population. Studies such as Sahgal et al. (2007) and Fatima et al. (2019) provide foundational knowledge to guide the development of region-specific protocols, emphasizing the importance of local control with acceptable toxicity and the comparative efficacy of SRS and SRT [17,27]. The ongoing assessment of treatment effectiveness, coupled with a deep understanding of local patient demographics and tumour characteristics, will be crucial for optimizing CyberKnife utilization.

Expanding Access and Awareness

Expanding access to CyberKnife technology in the UAE and the Middle East involves not just the acquisition of the technology but also raising awareness among healthcare professionals and patients about its benefits. Educational initiatives could focus on the versatility of CyberKnife in treating a wide range of meningioma types, including atypical meningiomas and large cranial base tumours, as evidenced by Choi et al. (2010) and Tuniz et al. (2009). Moreover, the comparative analyses provided by Fatima et al. (2019) highlight the importance of informed decision-making based on a comprehensive understanding of the risks and benefits associated with different radiosurgery options [17,29,30].

Future directions

Integrating CyberKnife technology into the UAE's healthcare landscape presents an exciting frontier for cancer treatment, particularly for meningiomas where precision and safety are paramount. Continued research and adaptation of global best practices to the local context will be key to unlocking the full potential of this technology. Collaborations between international experts and regional medical centres can facilitate knowledge exchange, ensuring that patients in the UAE and beyond have access to world-class care. The insights from the referenced studies form a robust foundation for such advancements, guiding the region towards a future where radiosurgery becomes a cornerstone of meningioma management. For physicians practising in the Middle East, particularly in the UAE, a comprehensive review of CyberKnife technology is essential. Given its recent introduction in the region, such a review is critical in assisting radiation oncology teams in understanding the efficacy and safety of this advanced robotic technology for patient treatment [45,46]. This insight is particularly relevant for managing meningiomas, where CyberKnife's precision and adaptability could offer significant benefits.

Limitations

Firstly, the inherent design of many retrospective studies raises concerns about potential biases, including selection and recall biases, which could impact the generalizability of the results. Secondly, the heterogeneity in treatment protocols, including differences in radiation doses, fractionation schedules, and radiosurgery platforms (e.g., CyberKnife vs. Gamma Knife), complicates direct comparisons across studies and limits the ability to draw definitive conclusions regarding the optimal treatment strategy. Additionally, the variability in follow-up durations across studies introduces challenges in assessing long-term outcomes and late radiation effects, which are critical for fully understanding the risk-benefit profile of SRS/SRT. Moreover, many studies lacked a control group, relying instead on historical control rates for comparison, which may not accurately reflect contemporary treatment outcomes. Lastly, the specific patient populations, tumour types, and locations included in these studies may not represent the broader population of patients with brain tumours, thereby limiting the applicability of these findings to all clinical scenarios.

## Conclusions

The synthesis of evidence from 21 studies affirms the integral role of CyberKnife radiosurgery in the modern management of meningiomas, spotlighting its efficacy, safety, and positive impact on patient outcomes. This treatment modality is a significant advancement, especially for patients contraindicated from surgery or whose tumours are in anatomically challenging positions. As the radiation oncological communities around the globe continue to explore and refine the applications of CyberKnife radiosurgery, this modality is anticipated to remain at the forefront of minimally invasive tumour management strategies, ultimately enhancing the therapeutic landscape for patients with meningiomas.
